# Preparation and Characterization of Cellulose Acetate Film Reinforced with Cellulose Nanofibril

**DOI:** 10.3390/polym13172990

**Published:** 2021-09-03

**Authors:** Azelia Wulan Cindradewi, Rajkumar Bandi, Chan-Woo Park, Ji-Soo Park, Eun-Ah Lee, Jeong-Ki Kim, Gu-Joong Kwon, Song-Yi Han, Seung-Hwan Lee

**Affiliations:** 1 Department of Forest Biomaterials Engineering, Kangwon National University, Chuncheon 24341, Korea; azeliacindradewi@gmail.com (A.W.C.); pojs04@kangwon.ac.kr (J.-S.P.); laa3158@kangwon.ac.kr (E.-A.L.); panda20@kangwon.ac.kr (J.-K.K.); 2 Institute of Forest Science, Kangwon National University, Chuncheon 24341, Korea; rajkumar.pgcb@gmail.com (R.B.); chanwoo8973@kangwon.ac.kr (C.-W.P.); gjkwon@kangwon.ac.kr (G.-J.K.); songyi618@kangwon.ac.kr (S.-Y.H.); 3 National Institute of Forest Science, Seoul 02455, Korea; 4 Kangwon Institute of Inclusive Technology, Kangwon National University, Chuncheon 24341, Korea

**Keywords:** cellulose acetate, cellulose nanofibril, eco-friendly plasticizer, film casting

## Abstract

In this study, cellulose acetate (CA)/cellulose nanofibril (CNF) film was prepared via solvent casting. CNF was used as reinforcement to increase tensile properties of CA film. CNF ratio was varied into 3, 5, and 10 phr (parts per hundred rubbers). Triacetin (TA) and triethyl citrate (TC) were used as two different eco-friendly plasticizers. Two different types of solvent, which are acetone and N-methyl-2-pyrrolidone (NMP), were also used. CA/CNF film was prepared by mixing CA and CNF in acetone or NMP with 10% concentration and stirred for 24 h. Then, the solution was cast in a polytetrafluoroethylene (PTFE) dish followed by solvent evaporation for 12 h at room temperature for acetone and 24 h at 80 °C in an oven dryer for NMP. The effect of solvent type, plasticizers type, and CNF amount on film properties was studied. Good dispersion in NMP was evident from the morphological study of fractured surface and visible light transmittance. The results showed that CNF has a better dispersion in NMP which leads to a significant increase in tensile strength and elastic modulus up to 38% and 65%, respectively, compared with those of neat CA. CNF addition up to 5 phr loading increased the mechanical properties of the film composites.

## 1. Introduction

Cellulose is a bio-based plastic polymer that can be derived from biomass. Thus, it is highly available. Cellulose-derived products such as cellulose acetate (CA), cellulose acetate propionate (CAP), and cellulose acetate butyrate (CAB) have been used in many industries such as automotive, biomedical, coatings, and packaging [1]. Among all the cellulose-derived products, cellulose acetate (CA) is the one that has been widely produced for thermoplastics. Cellulose acetate was successfully applied for the preparation of membranes in different membrane technologies [2], for wastewater treatment containing heavy metals or metallic ions [3,4], film packaging [5], in the textile industry [6], medical engineering [7], and in gas separation [8]. CA is prepared through acetylation of cellulose with an excessive amount of acetic anhydride by a sulfuric acid catalyst. The biodegradability of CA is based on its acetylation degree; CA with a low acetylation degree or substitution degree (DS: ≤2.5) can be degraded. CA is highly available, biodegradable, biocompatible, and relatively low-cost [9].

Considering these advantages, CA can be considered as a promising biodegradable plastic material that can serve an alternative for fossil-based plastic. However, CA has high stiffness and CA with DS around 2.5 has a narrow window between the melting point and the degradation temperature [10]. These properties are limiting the use of CA for wider applications. The use of a plasticizer can reduce the glass transition temperature of CA and also decrease the intermolecular force among the polymer chains, resulting in a softened and more flexible polymeric matrix [11].

Phthalate esters are the most common industrially used plasticizers for CA. However, they have been subjected to environmental scrutiny as a health threat, and thus there is now serious concern about their long-term use [12]. To improve the processing of CA, some studies have explored several eco-friendly plasticizers such as Triacetin (TA), triethyl citrate (TC), glycerol, and polyethylene glycol (PEG) [13,14]. These plasticizers are biodegradable, non-toxic, and have been approved as a plasticizer for many polymers. TA and TC are two of the most common eco-friendly plasticizers that have been used for CA. TA and CA share the same side functional groups: acetyl groups. TC is proved to be able to improve the stiffness and brittle of CA films [15,16]. Plasticizer addition into the CA matrix will decrease the tensile strength [16,17]. Therefore, it could impede the function of CA bio-plastics in specific industries. For this reason, the use of reinforcement or filler is needed to improve the tensile strength of CA [17]. Several studies have been conducted to improve the mechanical properties of CA using organo-clay, zeolite, microfiber, lignin, and nanocellulose as reinforcement [18,19].

Cellulose nanofibril (CNF) is one type of nanocellulose that contains long, flexible, and entangled fiber [20]. It has several advantages such as high tensile properties and high specific surface area, and being renewable and biodegradable [21]. CNF is considered as a potential reinforcing filler for manufacturing composites as it is renewable, lightweight, and cost effective [22,23]. Moreover, it also has high young’s modulus and tensile properties [24]. Despite these advantages, uniform dispersion of CNF in CA matrix is challenging. Dispersing CNF in the same organic solvent with CA is one method that can be taken to obtained good dispersion of CNF in CA matrix. Several studies have mentioned good dispersion of nanocellulose in some organic solvents such as dimethylsulfoxide (DMSO), dimethylformamide (DMF), and N-methyl-2-pyrrolidone (NMP) [25,26]. Berg et al., in 2007, found that NMP performs a faster dispersion process compared with DMF or DMSO [25].

In this study, CA was plasticized using two different kinds of eco-friendly plasticizers, TA and TC. Plasticized CA was then reinforced with CNF to improve the mechanical properties of the composite. CA film was prepared with a solvent casting method using two different solvents (acetone and NMP). The effect of plasticizer type, solvent type, and CNF amount on film properties were investigated. The novelty of the present work lies in the manufacture and comparison of the properties of new CA/CNF films using two different solvents so as to indicate the better solvent for film making.

## 2. Materials and Methods

Commercial CNF (3.9 wt% in water) was supplied by Cellulose Lab, Co., Ltd. (Fredericton, NB, Canada). Cellulose acetate (39.8 wt% acetyl content, DS 2.5, and average molecular weight Mn ~30,000), acetone (99.8% purity), triacetin (99.5% purity), and N-Methyl-2-pyrrolidone (NMP) were purchased from Daejung Chemical & Metals Co., Ltd. (Siheung, Korea). Triethyl citrate (99.0% purity) was purchased from TCI chemicals (Seoul, Korea).

CNF was solvent-exchanged from water to acetone or NMP using centrifugation at 18,000 rpm for 15 min followed by re-dispersion in the new solvent. Centrifugation was repeated at least 6 times to ensure all the water was exchanged.

**Scheme 1:**
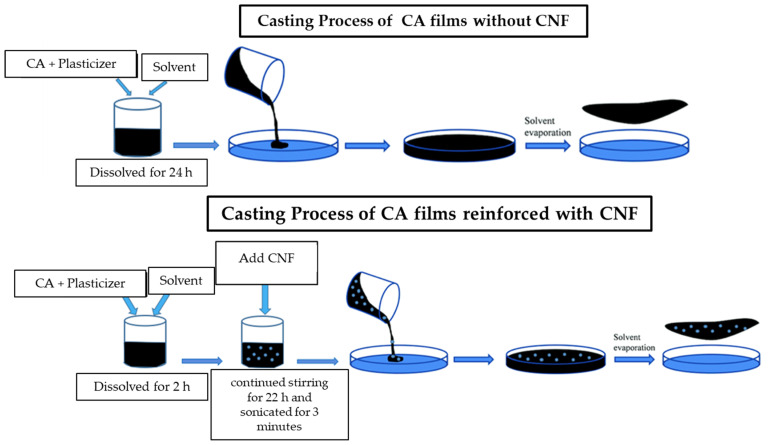
Schematic diagram of CA film preparation with and without CNF.

Films were prepared by solvent casting technique on a polytetrafluoroethylene (PTFE) dish (graphical illustration is shown in Scheme 1).

CA and plasticizer were dissolved in acetone or NMP solvent with 10 wt% concentration and then mixed for 2 h until homogenized. CNF in the same solvent was added with concentrations of 3, 5, and 10 parts per hundred rubbers (phr). The mixture was homogenized by magnetic stirring for 22 h continued with 3 min sonication. Then, plasticized CA/CNF solution was poured into the PTFE dish and solvent was evaporated by keeping at room temperature for 12 h for acetone and at 80 °C for 24 h for NMP. Neat CA and plasticized CA without CNF were also prepared using the same method with magnetic stirring for 24 h. Table 1 describes sample compositions for both acetone and NMP solvents.

Mechanical properties of the neat CA film, CA/TA, CA/TC, CA/TA/CNF, and CA/TC/CNF films were measured using a universal testing machine (UTM test one Model TO-102D, Siheung, South Korea) with a cross-head speed of 10 mm/min. A specimen span length was set at 30 mm. Samples were cut into dog-bone shapes as described by the American Society for Testing and Materials (ASTM D638) [27]. At least 5 specimens of each sample were tested, and the average values were taken. All of the tests were performed at 25 °C and 40% relative humidity.

The water absorption test was conducted based on the American Society for Testing and Materials (ASTM) D570 as the Standard Test Method for Water Absorption of Plastics [28]. Film specimens were dried in an oven for 24 h at 50 °C and then cooled in a desiccator, and immediately weighted to the nearest 0.001 g. The conditioned specimens were then placed in a container of distilled water at a temperature of 23 °C, resting on the edge, and were entirely immersed for 24 h. All the specimens were removed from the water at a time, and then surface water was wiped off with a dry tissue paper and they were immediately weighted to the nearest 0.001 g. The percentage of water absorption was determined by the following Equation (1):

(1) $$\mathrm{Water}\;\mathrm{absorption}(\%)=\frac{\left(\mathrm{wet}\;\mathrm{sample}\;\mathrm{weight}-\mathrm{dry}\;\mathrm{sample}\;\mathrm{weight}\right)}{\mathrm{dry}\;\mathrm{sample}\;\mathrm{weight}}\times 100\%$$

Thermal stability of neat CA, CA/TC, and CA/TC/CNF5 films was investigated using a TG analyzer (Q2000, TA Instruments Inc., New Castle, DE, USA) in the Central laboratory of Kangwon National University. The samples (5–10 mg) were heated on a platinum pan under a nitrogen atmosphere. The scanning temperature range was set from 25 to 700 °C, with a heating rate of 10 °C/min. The derivative TG (DTG) analysis was obtained by measuring the mass loss percentage with respect to temperature.

The viscosity of the CA/TC, CA/TC/CNF3, CA/TC/CNF5, and CA/TC/CNF10 samples was measured using a digital viscometer (DV-II+, Brookfield Engineering Laboratories, Middleborough, MA, USA). The temperature was maintained at 25 °C. The viscosity was measured with SC4-18 spindle at a range of shear rates from 0.4 to 132 s^−1^.

Fractured surfaces of CA/TC, CA/TC/CNF5, and CA/TC/CNF10 prepared in NMP samples were examined using a scanning electron microscope (SEM, S-4800, Hitachi, Ltd., Tokyo, Japan) in the Central Laboratory of the Kangwon National University. Samples were cryo-fractured using liquid nitrogen and directly used. Before the observation, the surfaces were sputter-coated with a platinum layer using a high-vacuum sputter coater (EM ACE600, Leica Microsystems, Ltd., Wtzlar, Germany) to avoid charging under the electron beam. The morphologies were observed at an accelerating voltage of 5 kV with a working distance of 8.5 mm.

CNF sample for SEM analysis was prepared with the following method. CNF suspensions were diluted to 0.1 wt% and then dispersed using an ultrasonicator (VCX130PB, Sonics & Materials Inc., Newtown, CT, USA) for 1 min. The suspensions were then filtrated on a polytetrafluoroethylene (PTFE) membrane filter with a pore size of 0.2 µm (ADVANTE^®^, Toyo Roshi Kaisha Ltd., Tokyo, Japan) using vacuum filtration. The filtrated CNF was then solvent exchanged from water to tert-butanol by immersing in tert-butanol for 30 min. The solvent exchange procedure was repeated 6 times to fully exchange water with tert-butanol. After solvent exchange, CNF was freeze-dried (FDB-5503, Operon Co., Ltd., Gimpo, Korea) at −55 °C for 2 h. The freeze-dried CNFs were placed on aluminum stubs and then coated with iridium using a high-vacuum sputter coater (EMACE600, Leica Microsystems, Ltd., Wetzlar, Germany). The X-ray diffraction (XRD) patterns of the films were recorded on an X-Ray diffractometer (X’pert PRO MPD, PAN analytical: Netherland; operating voltage, 40 kV; current, 30 mA) in the scan range of 10° to 35°. The surface functional groups were probed by Fourier-transform infrared (FTIR) spectroscopy (Frontier, PerkinElmer; UK) in the range of 400–4000 cm^−1^ at a resolution of 1 cm^−1^. Light transmittance of the films was measured on a UV-Vis spectrometer (OPTIZEN POP-V, Daejon, Korea) with a wavelength range of 400–800 nm. An empty cuvette was taken as a reference and the cut films were placed inside the cuvette and scanned.

## 3. Results

### 3.1. Mechanical Properties

**Figure 1:**
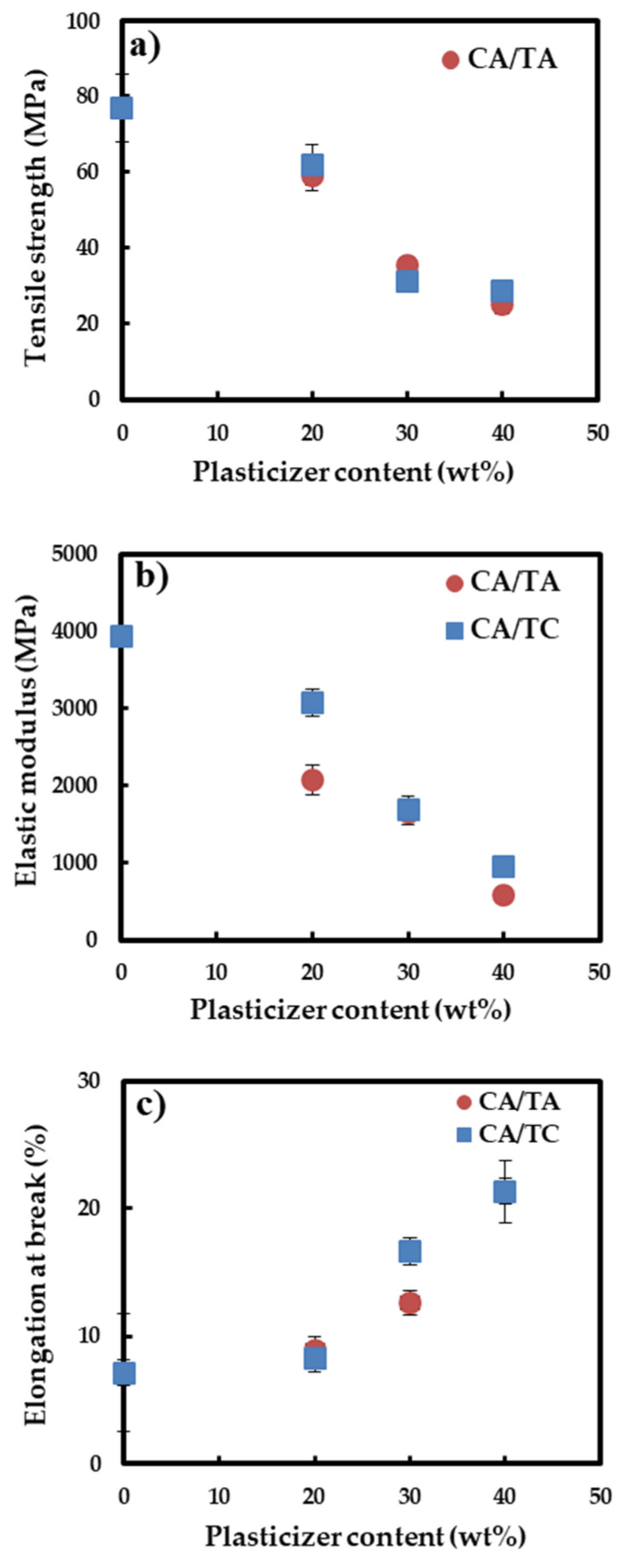
Tensile strength (**a**), elastic modulus (**b**), and elongation at break (**c**) of CA with TA and TC as a plasticizer in different ratios.

**Figure 2:**
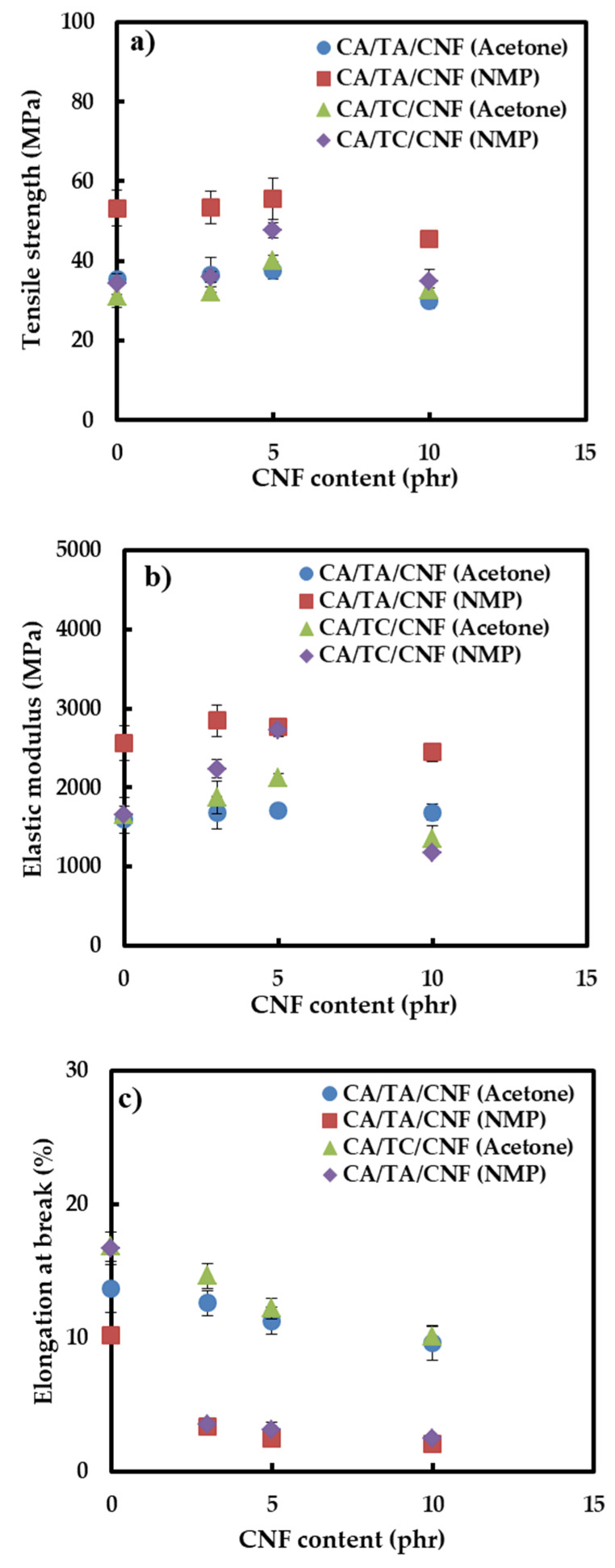
Tensile strength (**a**), elastic modulus (**b**), and elongation at break (**c**) of CA/TC/CNF and CA/TA/CNF in acetone and NMP solvent (Plasticizer content 30%).

Mechanical properties of CA/TC and CA/TA prepared using acetone as solvent at various ratios (60/40, 70/30, 80/20) were first investigated to choose the best plasticizer content. Figure 1 shows the mechanical properties of CA/TC and CA/TA. As shown in the figure, the addition of both TA and TC plasticizers decreased the tensile strength and elastic modulus of the CA film but increased its elasticity. This result is similar to previous reports. [15,16,18]. In the research conducted by Phuong et al. in 2014, 20% TA addition into CA obtained a tensile strength of 58.3 MPa, Elastic modulus of 3100 MPa, and elongation at break of 8.3%; when TA concentration increased to 30%, tensile strength decreased to 30.2 MPa, elastic Modulus decreased to 2100 GPa, and elongation at break increased to 11.6% [16]. Meanwhile, in the research conducted by Park et al., 2014, 20% TC addition into CA obtained a tensile strength of 70 MPa, Elastic modulus of 4100 MPa, and elongation at break of 8.5%; when TC concentration increased to 30%, tensile strength decreased to 61 MPa, Elastic modulus decreased to 2100 MPa, and elongation at break increased to 11.6% [11]. This can be explained because plasticizers (TA and TC) acted as a dilutor and lower the interaction of the molecules [29]. Based on this mechanical properties result, plasticizer content was fixed at 30% where elongation at break and tensile strength are in balance conditions. Both TC and TA were used in 30% content as a plasticizer for CA film reinforced with CNF. The effect of solvent type was also varied; acetone and NMP were used as two different solvents. Mechanical properties of CA/TC/CNF and CA/TA/CNF with varied CNF content in acetone and NMP solvent are shown in Figure 2. CNF addition up to 5 phr increased tensile strength and elastic modulus. Meanwhile, elongation break decreased with an increasing amount of CNF. The increase in elastic modulus is due to the presence of rigid nanoparticles from CNF. This shows that there is a stress transfer between CNF and CA matrix [30]. All samples with different plasticizer and solvent types showed the same patterns in mechanical properties. Similar results were also reported in a previous report by Leite et al., 2016, where 10% and 15% addition of CNC into CA increased elastic modulus up to 5% and 15% [30]. Research conducted by Azeredo et al. 2010 reported that 10% CNF addition into chitosan increases tensile strength up to 16% [31].

**Figure 3:**
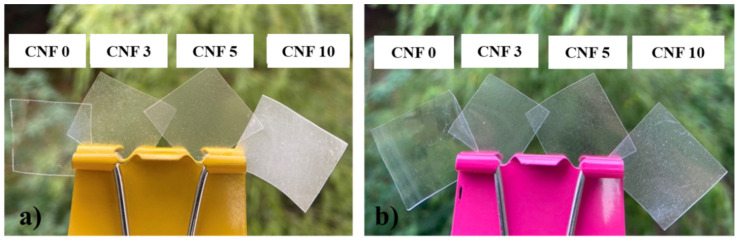
Digital photographs of plasticized CA/CNF films prepared in acetone (**a**) and NMP (**b**).

Compared with plasticized CA without CNF, CA/TC/CNF in NMP solvent has shown the highest improvement in both tensile strengths from 34.2 MPa to 47.6 MPa (38%) and elastic modulus from 1649.1 MPa to 2722.9 MPa (65%). This is probably because of good dispersion of CNF in NMP, which is also evident from the better transparency of CA films (Figure 3).

### 3.2. UV-Visible Spectroscopy

**Figure 4:**
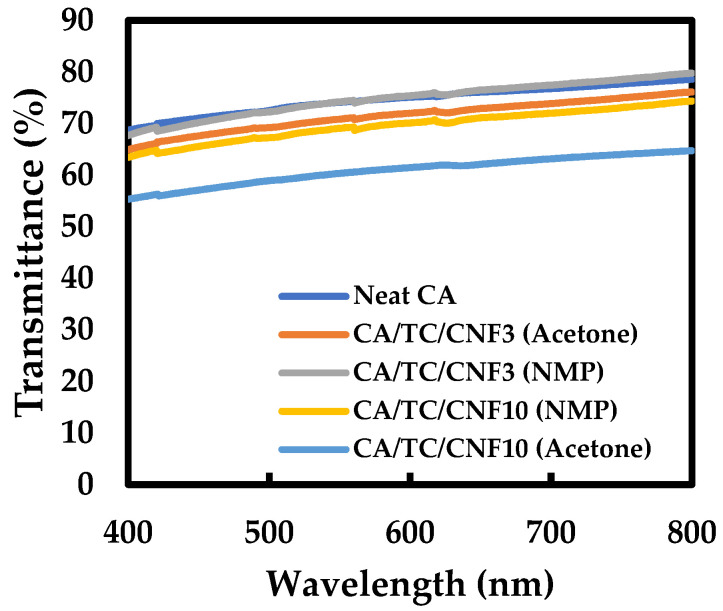
Visible light transmittance of CA films containing different ratios of CNF prepared using acetone and NMP solvent.

Good transparency is one of the advantages of CA. Because of its high transparency, CA is used for several applications such as high-quality frames for sunglasses, personal protective equipment, and sports goggles [32]. Therefore, when reinforcement is added into CA, it is expected that the transparency of CA could still be maintained. According to this result, NMP has become a possible alternative solvent to CA reinforced with CNF film for plastic application. Both TA and TC are clear colorless, thus the addition of plasticizers is not affecting the transparency of CA. UV-Visible spectroscopy was used to further compare film transparency. Figure 4 shows transmittance values of 68.7%, 64.9%, 67.7%, 63.4%, and 55.3% for neat CA, CA/TC/CNF3 (Acetone), CA/TC/CNF3 (NMP), CA/TC/CNF10 (NMP), And CA/TC/CNF10 (Acetone), respectively. Compared with acetone, CA film prepared with NMP has better transparency due to better dispersion of CNF in NMP than in acetone. This result is also supported by the tensile properties result where CA film prepared with NMP had higher tensile properties compared with CA film prepared with acetone.

### 3.3. Thermal Stability

**Figure 5:**
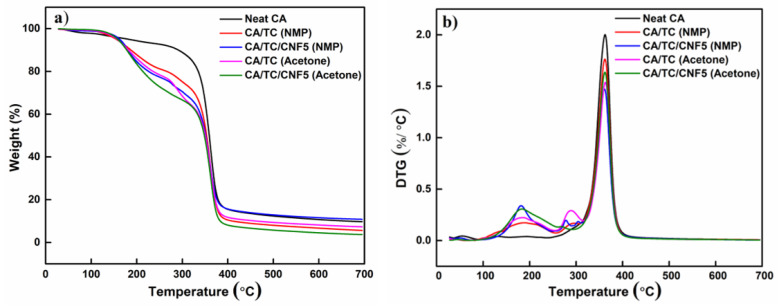
TGA (**a**) and DTG (**b**) graph of neat CA, CA/TC, and CA/TC/CNF5 in both acetone and NMP solvent.

Figure 5 shows the thermal degradation behavior of Neat CA, CA/TC, and CA/TC/CNF5 in both acetone and NMP solvent. Thermal degradation behavior was investigated by Thermogravimetric analysis. Neat CA film showed two stages of degradation behavior. The first stage at 100 °C can be attributed to the evaporation of water. The second stage starting from 320 °C to 400 °C can be attributed to the thermal degradation of acetate functional groups and glucose rings in the polymer chain [12,33]. With the presence of TC plasticizer in CA film (CA/TC), thermal degradation behavior was changed from two stages to three stages. The first degradation stage corresponds to the evaporation of water, whereas the second stage at around 230 °C is attributed to TC degradation [9]. The third stage at around 350 °C to 400 °C is attributed to the degradation of CA. This indicates that there is a physical interaction between CA and TC [11]. CA/TC/CNF5 has shown similar behavior in thermal degradation with three stages of degradation. However, the first degradation temperature is lower than CA/TC film. This might be because the interaction between TC and CA was reduced with the presence of CNF. In terms of solvent variation, NMP showed a slightly higher thermal stability than acetone. This is probably because NMP has a higher boiling point compared with acetone.

### 3.4. Water Absorption

**Figure 6:**
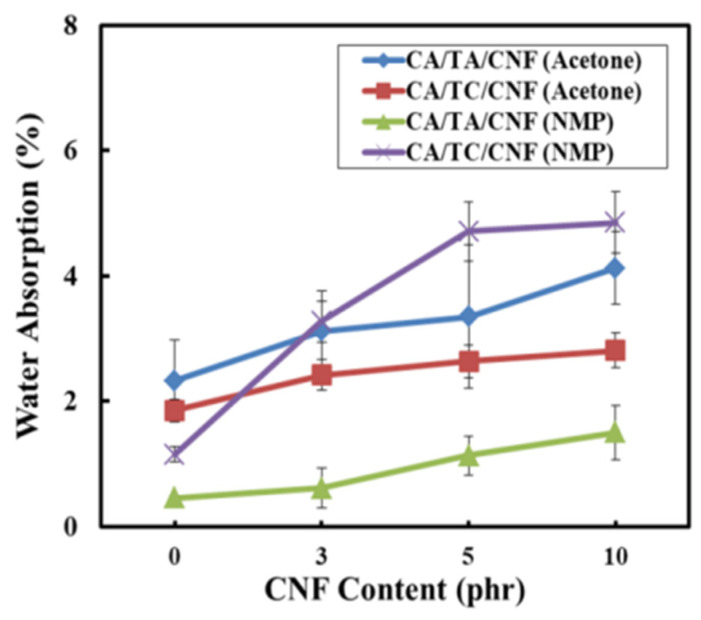
Water absorption of CA/TC/CNF and CA/TA/CNF with different CNF content prepared in both acetone and NMP solvent.

Water absorption is one of the important parameters for biodegradable plastic. Biodegradable plastic should have fewer water absorption properties for its functions. Water absorption was obtained from 24 h immersion in distilled water according to ASTM D570. The water absorption result is shown in Figure 6. All samples showed an increase in the percentage of water absorption with the addition of CNF. The increase in water absorption is related to the increased hydrophilic properties of the film upon adding CNF which is highly hydrophilic [34]. The water absorption percentage was relatively small (≤4.8%), and these results aligned with research conducted by Amri et al. 2021, where 0.5% CNF addition into waterborne polyurethane polymer had water absorption less than 3.5% [35]. This indicates the hydrophobic properties of the polymer still dominate the composite.

### 3.5. Viscosity

**Figure 7:**
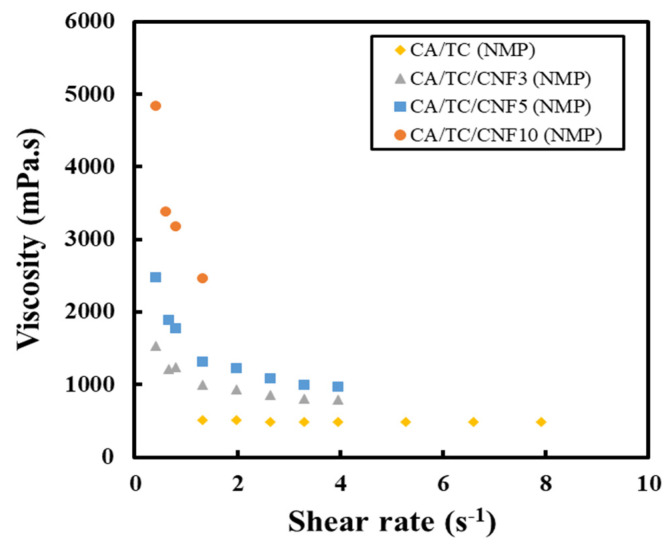
Viscosity of CA/TC and CA/TC/CNF with different CNF content in NMP solvent (Plasticizer content 30%).

Viscosity vs. shear rate curves are shown in Figure 7. CA fluid solution has shown typical curves of plastic fluid. The rheological behavior of other samples have shown a combination of non-Newtonian and Newtonian behavior. The viscosity of the solution was decreased at a lower shear rate; however, it was starting to be constant at the higher shear rate. A similar trend was also shown in research conducted by Park et al., 2021, where CNF was added into alginate (AL) [36]. This happened because with increasing shear rate, the polymer molecules start to untangle from each other and start to align them in the direction of flow, resulting in a decrease in viscosity.

The effect of CNF amount on the viscosity of CA/TC/CNF in NMP solution is summarized in Table 2. The viscosity was increased with an increasing amount of CNF filler. The increase of viscosity upon the addition of CNF filler could indicate good dispersion of CNF in the CA matrix which leads to a good interaction between CNF and CA macromolecules [37].

### 3.6. Morphological Observation

**Figure 8:**
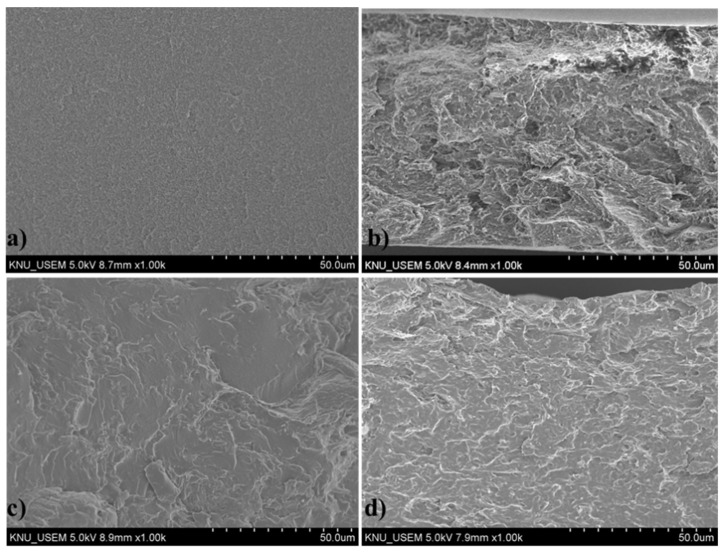
SEM images of the fractured surface of Neat CA (**a**), CA/TC/CNF5 (Acetone) (**b**), CA/TC/CNF5 (NMP) (**c**), and CA/TC/CNF10 (NMP) (**d**).

**Figure 9:**
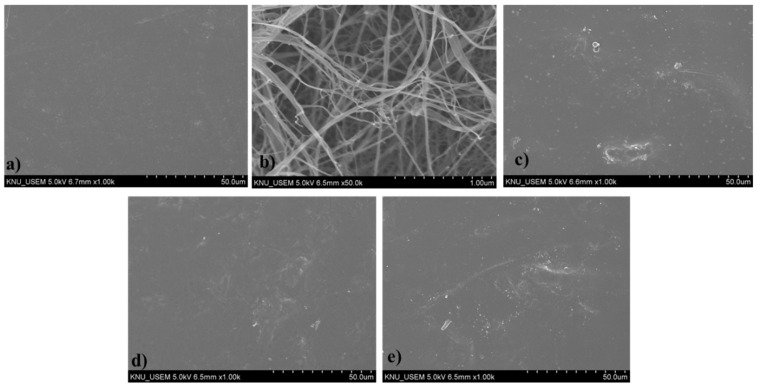
SEM images of Neat CA (**a**), CNF (**b**), CA/TC/CNF5 (Acetone) (**c**), CA/TC/CNF5 (NMP) (**d**), and CA/TC/CNF10 (NMP) (**e**).

Morphological observation from the fractured surface of CA film reinforced with CNF is shown in Figure 8. As shown in the images, the presence of CNF resulted in a drastic change in the structure of the CA composite. The CA composite with addition of 5% and 10% CNF had a rough structure in the fractured surfaces area. Meanwhile, the fractured surface of neat CA was smooth. The effect of solvent on the morphological characteristic was also studied, and CA/TC/CNF5 with NMP solvent showed less rough structure compared with acetone, even with a higher CNF content of 10%. SEM images of the surface area of neat CA, CA composite, and CNF are shown in Figure 9. As shown in the figure, Neat CA showed a very smooth surface area. Meanwhile, apart from CA, composite films exhibited a little aggregation of CNF, shown as white spots in the surface area, indicating the presence of CNF in CA film.

### 3.7. XRD and FTIR

**Figure 10:**
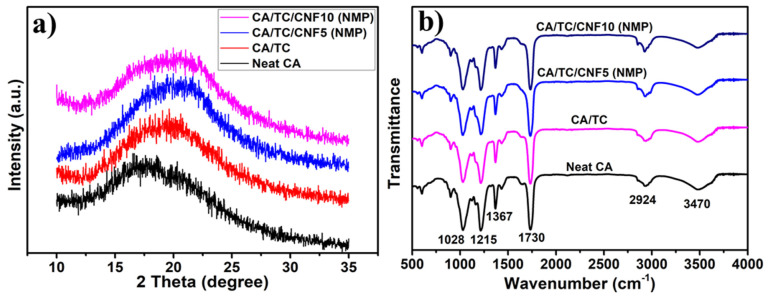
XRD patterns (**a**) and FTIR spectra (**b**) of Neat CA, CA/TC, CA/TC/CNF5 (NMP) and CA/TC/CNF10 (NMP).

XRD patterns of neat CA, CA/TC, CA/TC/CNF5, and CA/TC/CNF10 in NMP solvent are shown in Figure 10a. Neat CA exhibited weak cellulose peaks at 2θ = 18–22°. This is because CA has a substitution degree at ~2.5 where most of the hydroxyl group on cellulose are replaced with acetyl groups. As a result, intermolecular hydrogen bonding is absent or minimal, destroying the ordered crystalline structure [38]. Addition of TC caused no significant difference. However, the addition of CNF into CA matrix slightly increased the intensity of the peak in the 2θ = 18–22° range. No significant cellulosic peak was observed at 2θ = 18–22°, even at high CNF content (CA/TC/CNF10), indicating the CNF is uniformly distributed throughout the CA matrix. Figure 10b shows the FTIR spectra of neat CA, CA/TC, CA/TC/CNF5, and CA/TC/CNF10 in NMP solvent. For neat CA, the peak at 3470 cm^−1^ can be attributed to the O-H bond stretching, and the peak at 2924 cm^−1^ is for C-H stretching. The characteristic peak at 1730 cm^−1^ is assigned for carbonyl stretch of the acetate function and the peak at 1367 cm^−1^ can be assigned to C-H bending. Peaks at 1215 cm^−1^ and 1028 cm^−1^ can be ascribed to C-O stretch in C-O-H and in the C-O-C group respectively [1]. No significant changes in the peaks were observed after the addition of TC and CNF. Only a slight change in the intensity and shape of C-O peak at 1215 cm^−1^ was observed. This might be due to their uniform distribution and low loading content.

## 4. Conclusions

CA film was successfully prepared with CNF as reinforcement. Two different kinds of plasticizers, TA and TC, and two different kinds of solvents, acetone and NMP, were used. For both TA and TC, 30% content was chosen as the optimal condition for CA. When plasticizer content was more than 30%, tensile and elastic modulus properties of the CA film became unacceptably low. The presence of plasticizer in CA films changed the thermal degradation behavior of CA from two stages into three stages, also reducing film stiffness. The addition of up to 5 phr CNF into the CA matrix increased the tensile properties of the composite. However, the tensile strength was decreased with 10 phr CNF addition. The highest improvement in tensile strength and elastic modulus was gained by using NMP as a solvent because of better dispersion of CNF in NMP than in acetone. NMP was found to be a good solvent for the CA/CNF composite with higher tensile properties. Tensile strength and elastic modulus increased up to 38% and 65%, respectively. The use of CNF as reinforcement and TC and TA as eco-friendly plasticizer can expand the application of CA into wider applications, such as food packaging.

## Figures and Tables

**Table 1:** Sample compositions (applied to both acetone and NMP solvents).

Sample Name	Compositions (wt%) Weight			CNF (phr)
	CA	TA	TC	
CA	100	-	-	-
CA/TA	80	20	-	-
	70	30	-	-
	60	40	-	-
CA/TC	80	-	20	-
	70	-	30	-
	60	-	40	-
CA/TA/CNF3	70	30	-	3
CA/TA/CNF5	70	30	-	5
CA/TA/CNF10	70	30	-	10
CA/TC/CNF3	70	-	30	3
CA/TC/CNF5	70	-	30	5
CA/TC/CNF10	70	-	30	10

**Table 2:** Viscosity of CA/TC and CA/TC/CNF solutions in NMP with different CNF content and shear rate at a concentration of 10 wt%.

Sample Name	CNF Content (phr)	Viscosity (mPa.s)		
		SR 0.79 s^−1^	SR 1.32 s^−1^	SR 1.98 s^−1^
CA/TC	0	N/A	516	511
CA/TC/CNF3	3	1245	996	935
CA/TC/CNF5	5	1771	1314	1226
CA/TC/CNF10	10	3185	2463	N/A

## Data Availability

Data is available upon request from the corresponding author.
